# Older patients’ perspectives on the therapeutic relationship with young psychotherapists

**DOI:** 10.1371/journal.pone.0295834

**Published:** 2024-05-14

**Authors:** Annika Boschann, Hermann Staats, Silke Wiegand-Grefe

**Affiliations:** 1 Department of Psychiatry and Psychotherapy, UKE Medical Center Hamburg-Eppendorf, Hamburg, Germany; 2 Department of Psychology, MSB Medical School Berlin, Berlin, Germany; 3 Social and Educational Department, University of Applied Sciences Potsdam FHP, Potsdam, Germany; University of Verona, ITALY

## Abstract

The current demographic change means that young psychotherapists and older patients will increasingly come into contact. Unique for this constellation is the intergenerational therapeutic relationship, which forms the basis of psychotherapy, but has not yet been the focus of empirical research. This qualitative study provides preliminary insights into how older patients (aged over 65) experience and perceive the therapeutic relationship with young psychotherapists (aged in their mid-20s to mid-30s). We conducted semi-structured interviews with twelve older patients (8 women, 4 men) and analysed their data using the grounded theory approach. We found a connection between the type of transference a participant demonstrated and their biographical as well as social experiences, desires, and fantasies. Overall, a tendency to seek harmony was observed among the participants, which was reflected in their behaviour towards young psychotherapists: (a) conflict avoidance, (b) (fantasised) therapy discontinuation, (c) adaption/subordination, and (d) solidarity, support, and protection. Our findings demonstrated that various intergenerational transference phenomena, including the roles in which young therapists are perceived, are associated with certain particularities and challenges, such as the topic of sexuality. It can be valuable for young psychotherapists to become aware of a potential role reversal that may result in older patients trying to support them.

## Introduction

Worldwide, the proportion of persons aged 65 or over is projected to increase throughout this century. By 2050, persons aged 65 and older will make up approximately 16 per cent of the world population [1]. In Europe, persons in this age demographic currently comprise 18 per cent of the population, and this proportion is expected to increase to 25 per cent by 2050 [1]. As approximately 20–22 per cent of persons aged 65 and older meet the criteria for a psychological disorder or dementia (e.g., [2]), this demographic shift is important to consider from a mental health care perspective, especially since the increasing demand for psychotherapeutic treatment for the age group 65+ already seems to outpace the currently available capacity (e.g., [3]).

Considering these developments, age-appropriate mental health care for older adults will become more important [4]. Additionally, the demographic change means that young psychotherapists (generation G3) and older patients (generation G1) will increasingly come into contact. A central feature of any psychotherapy is the therapeutic relationship between therapist and patient. It has a significant effect on the treatment’s success, and “has long been recognized as an essential factor in the change process” (p. 1) [5]. The concept of the therapeutic relationship, also called the therapeutic alliance, has evolved over time and various empirical studies provide evidence of a moderate yet reliable association linking positive therapeutic outcomes to a good therapeutic relationship [6]. According to Horvath and Luborsky [7], the significance of the therapeutic relationship in psychotherapy can be attributed to Freud’s theorisation of relational phenomena between therapist and patient, which became known as “transference” and “countertransference”. Over time, both the concepts of “transference” as well as “therapeutic alliance” found relevance across diverse theoretical approaches to psychotherapy [8].

The therapeutic relationship plays an important role in psychodynamic and psychoanalytic therapeutic approaches based on their central assumption that the patient’s previous relationship experiences are unconsciously repeated and enacted within the therapeutic relationship. Consequently, reflecting the therapeutic relationship and analysing unconscious transference phenomena is essential for these approaches. Similarly, the therapeutic relationship is also relevant to consider for Cognitive Behavior Therapy (CBT) as every technique in CBT occurs within the context of the therapeutic relationship (e.g., [9]).

One unique characteristic of the constellation of young therapists and older patients is the intergenerational relationship dynamic is. Despite existing theoretical assumptions, this relationship dynamic has not yet been the focus of empirical research. While some preliminary insights into how young psychotherapists experience and perceive older patients were provided by Boschann et al. [10], a detailed exploration of the perspective of older patients concerning their young psychotherapists is still lacking. In particular, the therapeutic relationship between older patients and young psychotherapists requires further research as it forms the basis of psychotherapy and has a significant effect on the treatment’s success.

### Present study

This qualitative study aims to gain a deeper understanding of the experiences of older patients (aged over 65) who were treated by young psychotherapists (aged in their mid-20s to mid-30s). Particularly, this study intends to explore how older patients experience the intergenerational therapeutic relationship, which includes interpersonal behaviour and transference phenomena [cf. 11]. In this context, it should be noted that the term “transference” has developed considerably over time. While Freud initially viewed transference as an obstacle, he subsequently began to perceive transference as an “essential tool of the analytic process, observing how the patient’s relationships to their original objects were transferred to the person of the analyst” (p. 474) [12]. Since Freud (1905) [13] first indicated transference as essential to the analytic process, the term has been perceived differently depending on the respective analytical viewpoint. In this study, we draw on an extended conception of transference by Sandler, Dare and Holder (p. 43) [14]:

Transference […] can be taken to include the unconscious (and often subtle) attempts to manipulate or to provoke situations with others which are a concealed repetition of earlier experiences and relationships. […] Transference elements enter to a varying degree into all relationships, and these […] are often determined by some characteristic of the other person who (consciously or unconsciously) represents some attribute of an important figure of the past.

The primary research questions that guided this study were: How do older patients experience themselves, their young psychotherapists, and the therapeutic relationship? What are the characteristics, and challenges that can be derived as clinical implications?

By conducting semi-structured interviews with older psychotherapy patients (aged 65–86) and applying the grounded theory approach, an initial preliminary model of the experiences of older patients who are treated by young psychotherapists was developed. This study aims to deepen our understanding of the intergenerational therapeutic relationship, including transference phenomena and behavioural patterns exhibited by older patients, and thereby support young psychotherapists who work with older patients.

## Method

### Ethics

This study was approved by the Ethical Committee of MSB Medical School Berlin (MSB-2020/35) and complies with the principles expressed in the Declaration of Helsinki. Informed written consent for participating in the research and for saving and storing the voice recordings was obtained from all participants. The interview transcriptions omit any identifying personal information such as names and places. To safeguard the participants’ privacy, all personal details have been anonymised and pseudonyms were used.

### Participants

The participants were recruited via young therapists working in psychotherapeutic training institutions, geropsychiatric clinics, and medical practices in Berlin, Germany. The psychotherapists were asked to share information on the study with their older patients, some of whom subsequently voluntarily and independently contacted the first author (AB). AB did not engage in a therapeutic relationship with any of the participants. Overall, the sample consisted of 12 older patients (8 women, 4 men) aged between 65 and 86 years old (average age of 74 years). These participants met the inclusion criteria of being at least 65 years old and having undergone psychotherapeutic treatment with one or more young psychotherapists (licensed or in training, aged mid-20 to mid-30). Psychotherapy was ongoing or completed within the last 1.5 years; both psychodynamic and behavioural treatments were included. The details of the participants are shown in Table 1. All participants were White German citizens.

**Table 1:** Participant demographic data.

Participant	Age	Sex	Therapeutic Approach	Grandchildren	Marital Status
Phil	71	Male	Day-care hospital (BT)	No	Married
Grace	76	Female	Day-care hospital (PT) Outpatient (PT)	Yes	Married
Harriet	83	Female	Outpatient (BT)	Yes	Married
Richard	79	Male	Outpatient (BT)	No	Single
Daisy	71	Female	Outpatient (BT)	Yes	Divorced
Martha	74	Female	Outpatient (BT)	No	Divorced
Rosie	69	Female	Outpatient (PT)	Yes	Married
Mary	86	Female	Inpatient clinic Day-care hospital (PT)	No	Single
Jack	72	Male	Inpatient clinic Day-care hospital (BT)	No	Divorced
George	65	Male	Day-care hospital (PT)	Yes	Married
Sue	69	Female	Inpatient clinic Day-care hospital (PT)	Yes	Married
Violet	80	Female	Outpatient (BT)	Yes	Divorced

*Note*. All demographic data are self-reported. BT = behavioural therapy; PT = psychodynamic therapy. All names are pseudonyms.

### Research team

The research approach was guided by psychodynamic and geropsychological perspectives. Since the researchers have a psychodynamic professional background, psychodynamic perspectives guided the formulation of the research question, the coding process and the interpretation of the results. The initial research team consisted of the three authors: a female 31-year-old PhD candidate and clinical psychologist in psychodynamic training with therapeutic work experience in geriatric settings, and two professors in clinical psychology both of whom are experienced psychodynamic psychotherapists. For the coding process, the research team was expanded to include two additional female 31-year-old clinical psychologists, for whom external funding was provided by the German Psychoanalytic Association. Both have inpatient or outpatient work experience with older patients. One of them is a doctoral candidate in psychodynamic training while the other works as a counselling psychologist. Additionally, we held regular group discussions with other qualitative researchers from an academic research group from the University Medical Center Hamburg-Eppendorf, Germany to develop the tentative theoretical framework.

### Researcher reflexivity statement

Qualitative research is a form of research in which the researcher or designated coresearcher collects and interprets data, making the researcher as much a part of the research process as participants and the data they provide. (p. 4) [15]

This quote aligns with our understanding of qualitative research principles and resonates with our role as researchers throughout the research process. Our research was guided by a constructivist-interpretivist paradigm. The core assumption was that we cannot assume or describe an objective reality void of our perceptions, values, and experiences (cf. [16]). Aligned with this perspective and following Corbin and Strauss [15], we argue that the formulation of concepts and theories stems from our role as researchers constructing and interpreting the stories, experiences, and narratives of the participants. In line with the constructivist-interpretivist paradigm, we believe that the research process is influenced not only by the participants, but also by the researchers’ values, interpretations, and personal experiences. Therefore, we cannot describe or delineate an objective reality; instead, we need to recognise and reflect upon the fact that reality is also shaped by the researcher’s perceptions, values, and experiences (cf. [16]). Consequently, we were mindful of the fact that the interviewing researcher is a young therapist herself and that the data was coded by young female researchers. To reduce personal subjective assumptions and remain receptive to the experiences and viewpoints of the participants, we engaged in extensive discussions and critical reflection. Thus, regular team meetings were used to discuss views, perceptions, personal expectations, professional experiences with older adults, as well as possible counter-transferences, and their potential influence on the research process. Following each interview, the interviewer compiled a postscript to document personal observations of the conversational atmosphere, interpersonal dynamics, evoked emotions and associations. Given the considerable age gap between patients and therapists, we initially expected to mostly find evidence of a grandchild transference. However, as the research analysis progressed, our perspectives on how older patients experience and perceive young psychotherapists became more multi-faceted.

### Procedure

#### Interviews

The interviews were conducted by the first author (AB) during the period spanning July 2020 to September 2021 and audiotaped. Using a semi-structured interview, participants were asked to reflect on their experiences with young therapists. We used a problem-centred interview approach to capture and compare individual experiences as well as the subjective perceptions of the participants [17]. The interview guide (see S1 File) included four dimensions with open-ended questions concerning the participants’ experiences of the therapeutic relationship, transference phenomena, and both opportunities and challenges of working with younger therapists. In each interview, participants were encouraged to openly share their perspectives. Coverage of all dimensions and adjustments to allow for a coherent flow of the conversation were considered. Based on the grounded theory approach, we used theoretical sampling and collected new required data in parallel to the data analysis. The sample compromised of 12 participants as theoretical saturation was observed after completing the 12th interview. At that point, further data collection was unlikely to add any substantial new insights or information to the comprehension of the tentative theory (cf. [18]).

The interviews lasted about 80 minutes on average. Because the interview period took place during the Covid-19 pandemic, ten interviews were conducted at the participants’ personal residences and two interviews took place at psychotherapy training institutions, depending on the individual wishes and needs of the participants.

#### Grounded theory data analysis

The methodology of Grounded Theory, initially formulated by Glaser and Strauss (1976), centres around an inductive analysis process which means that the constructed theory is derived in the data. At its core is the manual coding, categorisation, and comparison of collected data [18]. Rather than testing a theory against the data, the theory is inductively generated from the data, with data collection and analysis as a joint task [19,20]. Different approaches to Grounded Theory have evolved over time. For our research, we followed the constructivist approach as put forward by Corbin and Strauss [15].

The interviews were transcribed verbatim and the analysis was conducted using the MAXQDA 2020 software (VERDI GmbH) as well as manual processing of the manuscripts. Given the relatively small existing body of research on this topic, a qualitative theory-generating approach was chosen. The grounded theory and an interpretive design were used to gradually deepen our understanding of the participants’ individual experiences and behaviours (cf. [21]). This approach is characterised by a process of discovery, induction, constant revision, and comparison of the interview material (e.g., differences or general patterns) [18].

We analysed both the manifest, explicit data material, as well the latent, preconscious content of the interviews. We assumed that the latter would most likely provide insight into the participants’ unconscious experiences. Based on the grounded theory approach, the following three systematic steps formed the basis of the data analysis for each interview: open coding, axial coding, and selective coding [15]. No predefined sequential order was followed when analysing interviews and coding the data. Throughout the analysis, we produced memos to collect our reflections, questions, and ideas on how the codes were connected to form categories. The data analysis was performed as described in Boschann et al. [10]. Briefly, the relations of the categories were differentiated using information derived from our memos to conceptualise a tentative theory. Open coding was used to derive inductively formulated codes that were subsequently categorised, after which axial coding was applied to develop a category system by reorganising the data. The core category, which is related to all main categories, was identified during selective coding.

In our data analysis, we adhered to the constant comparison method, also referred to as “comparative analysis”, which serves as a strategic approach for theory generation (p. 39) [22]. Following the initial segmentation of data into manageable units, we assessed these units for shared patterns and distinctions. Those units displaying similarities were grouped together under the same conceptual headings. As the analysis process advanced, these concepts were grouped into overarching themes and categories, which were then refined in relation to their distinct attributes and dimensions. Finally, we integrated various categories around a core category (i.e., the primary theme). This core category, combined with the properties of the other categories, formed the basis of our Grounded Theory [15].

Each interview was initially analysed separately by two researchers while the other team members subsequently reviewed the codes and categories of each interview at a later stage. To minimise interpretation errors, we compared the interim results, developed the tentative theoretical model and discussed our findings and the researchers’ process of analysis in regular team meetings [23].

## Results

The participants’ description of their relationships with the young therapists or rather, the therapists’ perceived roles, varied across participants. The analysis of the interviews revealed a connection between the type of transference a participant demonstrated and their biographical and social experiences, which were linked to their desires and fantasies. Furthermore, a common feature of all interviews was the participants’ tendency to seek harmony (core category) which was demonstrated in their behaviour towards young therapists (a. conflict avoidance, b. (fantasised) therapy discontinuation, c. adaption/subordination, d. solidarity, support, and protection). It should be noted that we refer to self-reported behaviours described by the participants. In the following, the individual categories are described in more detail by using illustrative examples from the interviews, whereby pseudonyms are used for each participant to protect their anonymity.

### Transference and perceived role of the therapist–experiences, desires, and fantasies

Several participants found it important that young therapists took them “seriously” and treated them “as equals” (e.g., Mary). Concerning this aspect, some participants indicated that it was important to them to not feel old when they were with a young therapist. A few participants reported feeling that they “no longer had a role to play in the work environment” or that they had concerns about being “too old” for psychotherapy (e.g., Rosie). However, Jack reported that young therapists “never made him feel that psychotherapy was no longer worthwhile because of his age. Instead, Jack felt that his young therapists saw him “as a well-rounded and valuable individual” and that he was not “just written off as a granddad”. Martha liked that her therapist was compassionate, but “did not sugar-coat” things because of her age and instead treated her “completely normal, like other patients”. Similarly, Rosie explained:

That was another thing I liked. She [the therapist] never gave me the feeling that I was old. I really felt that I was on the same level as her. I just really didn’t feel old. And I felt like she took me seriously. I also felt like there was no judgment from her.

Overall, the findings showed that the way participants perceived young therapists varied. The analysis identified different types of transferences or roles in which the young therapists were perceived, some of which appeared in combination. The following transferences/ roles were observed: child (son/daughter, son-in-law/daughter-in-law), grandchild, erotic love transference, as well as perceptions of authority.

Further analysis showed that the participants’ biographical and social experiences as well as their desires and fantasies played a role in how the young therapists were perceived. Some participants described relationship experiences from their own childhood and adolescence, while others mentioned later experiences with younger people in their family (e.g., children and grandchildren) or at work. Some participants referred to personal experiences of being a younger adult and compared themselves to their therapists. For example, Rosie reflected on how, when she was young, she used to feel anxious during her own professional training. She compared this to the confident, competent appearance of her young therapist. Similarly, Martha linked her need to “always be perfectly prepared” in her previous job to the professionalism and perfectionism of her therapist and highlighted this as a commonality between them. Some participants also recounted previous experiences with psychotherapy (e.g., with older therapists in the German Democratic Republic, GDR) and the general medical-psychiatric care system and related this to their (mostly positive) experiences with young therapists. Moreover, the analysis of the manifest as well as the latent content of the interviews showed that, the participants expressed certain desires (e.g., desires for a partner–Richard) and fantasies (e.g., imagining a “replacement grandchild”–Martha). Some of the transferences/ perceived roles of the young therapists identified during the analysis and the experiences, desires, and fantasies associated with them are outlined below.

#### Martha–outpatient example of a positive child transference

Martha, a 74-year-old woman, came to see her young therapist due to depressive symptoms and conflicts with her daughter. There was little contact with her daughter and her desire for a grandchild remained unfulfilled. Martha described initially feeling sceptical because of the therapist’s gender and young age:

The problem was that I had always had a female therapist and was looking for one again but then it turned out to be a man this time. And then the other problem was that he was much, much younger than me. He could have been my son.

However, she described how this feeling quickly subsided in the course of the therapy, which became a very positive experience. Martha emphasised that her therapist had always been “very professional”, but had once “crossed a line” when he told her he had recently become a father. Following this, Martha developed considerable interest in her therapist’s baby and explained: “I have the feeling that he could have been my son, so I was obviously interested in the baby, you see?” Martha would have been “extremely pleased if he had told [her] a bit about his child” and often asked her therapist for baby photos. At first, he had shown Martha baby photos, but when his reaction seemed distant at a later time, she strategically waited for several sessions before asking him again, to avoid potentially annoying him. Martha had had a “replacement grandchild” before and referred to her therapist’s baby in the same way: “I had the same feeling again. He could be my son and now there is a grandchild, as it were. Because I don’t have one”. Martha had “imagined her therapist as a father” and imagined “how lovingly he interacted with the baby.” Martha developed fantasies of “meeting him outside with the pram”:

If he had said ‘I am going to be in the park on Sunday afternoon’, I probably would have been in the park as well and would have bumped into him by accident (laughs).

Martha stated that she did not discuss “sexual issues” with her therapist because she is “probably too old-fashioned”.

#### Daisy–outpatient example of a negative child transference

Daisy was a 71-year-old woman, suffering from anxiety and post-traumatic stress disorder. As a child, she was sexually abused by her father and was also abused by a male therapist in a children’s home. As an adult, Daisy was a political prisoner in the GDR and her baby was taken away from her. She often referred to her young male therapist as “the boy” and stressed that he was “pleasant” but “like a child” to her. Their first meeting already got off to a “bumpy” start and Daisy had difficulty telling him about her traumatic experiences. She recalled it being “unbelievably difficult” to tell a young therapist about the abuse she suffered and that she had the impression that he felt “uneasy”. Daisy stressed: “He was really sweet. He would have made a lovely son-in-law. But not my therapist.” She indicated that she finds it “easier” to speak to older people about her experiences and explained that the reason she felt “sceptical and distant”, “definitely had something to do with [his] age. He might be very competent, I’m not denying that, but he was just confounded by my experiences, poor kid.” Daisy also recounted that the therapist seemed “disbelieving” when she told him about the sexual and physical abuse and her experiences in prison. She related this to the young age of the therapist, as well as their different socialisation experiences:

He probably had no idea about the sort of things I experienced (…) What I experienced is so unbelievable. I grew up in the GDR and even an East German would have difficulties believing the things I went through. A West German would have no idea.

After having “this feeling”, Daisy stopped seeing this therapist: Something happened then which happened to me a lot as a teenager and a child: I had the feeling he didn’t believe me. That is a very, very hard feeling for me to have.

When I had this feeling, it was over for me. I no longer went to see him.”

Afterwards Daisy switched to a young female therapist with whom she felt a “very strong relationship of trust.” This young therapist was “really very empathetic, really sweet” and they would tailor the therapy sessions to her needs. The relationship that developed between them was “almost like a friendship.” Nevertheless, Daisy would not talk about sexuality with young therapists.

#### Rosie–outpatient example of a grandchild transference

Rosie, a 69-year-old woman, came to see a young therapist because of a psychosomatic disorder and anxiety symptoms with depressive components. Rosie had “relived traumatic experiences after surgery that threw [her] off track” and was “very happy and grateful” to have a place with her young therapist. Rosie “immediately saw [her] granddaughter sitting in front of her” who had “also wanted to enter this profession”. The “chemistry was right from the start” and it was “love at first sight”, something she had only experienced with her husband before. Rosie had “known it would work out with her [the young therapist]” from the first moment. According to Rosie, a deep bond of trust developed “very quickly” and the therapist is “an important support and was always there” for her. Although her expectations had been low due to past negative experiences of therapy with older therapists in the GDR, this time she did not feel “any fear or inhibition” and stated “I don’t feel afraid of being hurt in some way when I’m with her. (…) I feel good with her.” The relationship with her young therapist is “harmonious” and she encourages Rosie in what she has “already achieved and accomplished.” In comparison, Rosie had “suffered” through “a lot of conflict and a lot of confrontation” in her earlier therapies. Rosie likes the fact that her young therapist usually emphasises her strengths and successes. Rosie described a strong relationship with her granddaughter, with whom she is very close (“she’s my sweetheart”). She spoke about having an affinity for young people and enjoying being with them: “I just like young people. I don’t really like spending time with older people (laughs).” Rosie was pleased that both her granddaughter and her therapist were interested in her life “despite being an older woman” and that both of these relationships are harmonious. Sometimes her stories about her granddaughter and therapist became mixed up during the interview so that Rosie would laugh and clarify: “My granddaughter! I am talking about my granddaughter!” Rosie admitted to not wanting to talk to her therapist about her sexuality, although this played a major role for her: “That is an impediment if I’m being honest. I couldn’t talk to her about that.” She added that she would not talk to her granddaughter about her sexuality either. When her youngest son and his partner broke up, Rosie also imagined her therapist as her daughter-in-law.

#### Richard–outpatient example of an erotic love transference

Richard, a 79-year-old man, suffers from depressive moods, panic attacks, and anxiety about his pacemaker. He mentioned that he often feels lonely and longs to find a partner. Richard spoke enthusiastically about his young therapist and emphasised how attractive she was. He emphasised their “wonderful talks”: “It is so great. I really enjoy speaking to her and having a conversation. That’s something I miss. I miss that at the moment.” From his first meeting with his young therapist, he said, he was “gobsmacked by this woman” and described:

Such a lovely, sympathetic, pretty woman. (…) Such a charming and attractive woman, that was the first impression. Moreover, we have such nice conversations, such nice chats.

Richard related that he also meets “other women as well” but that his therapist is “special” and “appeals” to him as a woman. Richard finds it difficult to leave when the sessions come to an end: “When I go home, I am only thinking of her, I always look forward to the next session.” Richard spoke of having given his therapist flowers and described the therapy process with the words “we have been together for a while now.” He admitted being attracted to her:

You always have to think about that a bit when you are an older man sitting opposite a young, pretty woman. (…) That’s just the way it is, these feelings, you can’t just dismiss them and say ‘No, I’m just a cold block of ice, aren’t I’?

Although Richard worries about the end of his life a lot, it “hasn’t come up in therapy”. Instead, he often talked about his search for a partner and would, for example, also bring newspaper clippings of partner adverts to the therapy sessions to discuss them.

### Tendency to seek harmony

The analysis identified a tendency to seek harmony as the core category which is related to the four subcategories outlined below. Overall, 11 participants saw working with young therapists as being fundamentally positive and enriching and most participants indicated that the therapy sessions did them good and helped them. Richard emphasised how important it is to him for the therapy to be harmonious (“calm and nice with a peaceful atmosphere”). Some participants described the atmosphere as being friendly or chummy, such as Sue, who said: “I like her [the therapist] a lot. She seems like she’s on the same level as me. I can talk to her as if she were a good friend of mine.” In general, Sue felt less inhibited with young therapists compared to older therapists, whom she found stricter and “more authoritative”. A few participants, such as Daisy, expressed fantasies of meeting in a more comfortable setting for “coffee and tea” to build up “trust” and “intimacy”.

Rosie emphasised that her desire for harmonious relationships had changed as she grew older. As part of that, she had moved on from having conflict-prone friendships and was glad that she no longer had to deal with difficult colleagues at work. She spoke about the rest of her life and explained that she did not want to “waste” the time remaining:

I don’t need that anymore. (…) I want to be healthy. I want to be able to enjoy the–hopefully many–wonderful years I have ahead of me in good health and I want to do everything to ensure that I can do that with what’s important to me now.

In the past, Mary also had had “disputes” both in friendships and at work, but now she said she is “too old” and found it “always important to get along well and not cause trouble”. Similarly, Grace linked her feeling of not wanting to get “annoyed or angry” or “hurt or challenged” by her therapist, to her old age (“maybe because I’m older”). She further explained how much she enjoyed the talks with her young therapist since she did not have “someone good to speak to at home”.

#### (a) Conflict avoidance

During the interviews, it was noticeable that some participants did not initiate discussions concerning conflict in the interviews. The majority of the participants emphasised how good the psychotherapeutic treatment and the relationship with their young therapists had been. When asked about possible difficulties or interpersonal tensions, most participants denied having had a conflict with their young therapists. For example, Richard stated that there had “never been any conflict at all” and it had “always been harmonious”. Harriet also vehemently denied experiencing any conflict with her therapist and stressed that her therapist “always did everything well”. Similarly, Grace explained that while there were “never any conflicts of any kind” with her young therapist, they “might have had constructive discussions sometimes.” Violet mentioned “sometimes having a conflict or a discussion” with her therapist, but “that everything was always back in balance” next session.

Analysis of the material for latent content also indicated conflict-avoiding behaviour among the participants toward young therapists. Richard, who suffers from a fear of death connected to his heart disease, did not raise this issue with his therapist. Instead, they would have “joyful and pleasant conversations” in which he spoke about his search for a partner. Similarly, Martha described keeping interpersonal conflicts out of the therapeutic relationship. For instance, although she was sometimes annoyed by her therapist, she never communicated her frustration and “practically always dealt with the conflict alone”. Instead, she praised her therapist in the next session for challenging her on purpose. When having a conflict with her young therapist, Violet thought to herself: “well, let him talk (laughs), at some point, this [conversation] will be over anyway.” She would become “completely quiet” and her therapist “senses that something is not right and changes the topic”.

#### (b) (Fantasised) therapy discontinuation

Daisy, whose experience with a young male therapist had been negative and involved interpersonal conflict, stopped her psychotherapeutic treatment as a result. She then found a young female therapist who was “really very empathetic [and] very sweet,” who gives her a lot of freedom and responds to her personal needs (e.g., a more flexible setting and frequency of the sessions). Daisy described “this current kind of therapy” as “absolutely perfect”. Some participants were hostile to potential conflict situations (i.e., having an argument) and threatened to discontinue their therapy as a result. Grace said: “To be perfectly honest, if I had noticed any kind of conflict at all, I may well have said goodbye.” She explained that she did not want to have any interpersonal conflicts with her therapist because she had enough conflicts “outside.” Similarly, Harriet said she would have had to “stop” if there had been a “bad atmosphere.” Mary also stated that if a conflict had developed, she would “probably have been done” with the therapy, while Violet reported she would have “dropped” therapy or considered a “change of therapists” if she did not feel comfortable with her young therapist.

#### (c) Adaptation / Subordination

Many participants described feeling a strong motivation to commence therapy, such as Grace, who explained she “really wanted” therapy instead of being “forced” to find it. Some participants made it clear how seriously they took their psychotherapy and that they were annoyed, for example, when other less motivated patients disrupted the group therapy session. Only a few participants spoke about resisting psychotherapy at the start. Phil explained that he had “quite considerably resisted” during the first few days in the day-care hospital and that he “hadn’t made things easy”. However, he had transformed into a patient who was very motivated and committed to participating and even had “fun” at the sessions. Many participants felt grateful and expressed a desire for their young therapists to help them, as illustrated by Rosie who emphasised that she would “never have got her life on track alone” and “often wouldn’t have made it”. Some participants said that they saw young therapists as being people worthy of respect and commanding authority (“They were authority figures for me”–Phil). For Jack, the young therapists were experts and that is why he “accepted their opinion regardless of their age (…) Because [he is] the one looking for help.” Relatedly, Richard commented that his therapist is “the person, who is there to help me” that he would “defer to her completely and expects her to recommend things,”

#### (d) Solidarity, support, and protection

On the one hand, many participants particularly focused on the skill level and professionalism of their young therapists, which they mainly attributed to the many years of study and training to become a therapist. Reflecting on her young therapist in training, Rosie mentioned that she did not notice “any differences from the ‘qualified therapists’” she had had in the past and stated: “I would have thought she was already a fully qualified therapist.” Martha also emphasised how professional her therapist was and that she had the feeling that “he had only prepared for me that day. He was completely focused on me and knew exactly what we had discussed in the previous session.” George referred to his therapist’s professional experience:

Obviously, she is very young, but she had already had experience in the day-care hospital (…) It wasn’t an issue for me because I really found her to be very, very competent.

On the other hand, most participants also described feeling a sense of solidarity with young therapists or feeling supportive or protective of them. Some participants emphasised that young therapists had a “difficult job” (Richard) and thus they showed compassion (“They don’t have it easy”–Grace). Rosie mentioned that she enjoys going to her young therapist, “because we need the next generation and that’s why it was important to me”. Relatedly, Violet explained that “young people have to be encouraged and have to learn somehow”. Phil had the “strong and distinct” feeling of being “in the same boat” as young therapists. In general, he felt a “need to help” young therapists and to “support” them in the group therapy sessions. He explained that he saw working with a therapist as a give and take: “Because they were nice to me, I was nice to them (…) That’s why I then wanted to help these therapists as well.” Violet described feeling overwhelmed by an exposure exercise but continued doing it for her young therapist anyway so as “not to disappoint” him:

He tries so hard and then I always think: ‘No, you have to pull yourself together.’ (…) When someone tries so hard and then the person, you’re trying so hard for just says: ‘No, I don’t want to.’ Well, that’s a crushing blow.

Additionally, Daisy described her own “uncertainty” concerning what she was allowed to tell young therapists. She was “definitely more cautious” and “more restrained” and suggested she did not want to “hurt” young therapists:

Someone is sitting there in front of you who could be your own child. My daughter has already offered to do “inner child work” with me 100 times, but no, never with my children. Never. The age difference makes me more cautious.

## Discussion

The study focused on older patients’ perspectives to gain clinical insights from their therapeutic experiences with markedly younger psychotherapists. In doing so, we aimed to explore how older patients experience and perceive the therapeutic relationship with young therapists. Our results should be understood as forming the basis of a preliminary conceptual model, which draws on the data of 12 older patients and thus is ongoing and provisional. Generally, the data shows that older patients view young therapists in different ways and describe individual therapeutic relationships with them. The findings highlighted that identification (personal experiences of being a younger adult) and previous object relationships, such as experiences with younger people in family contexts, play a fundamental role in the intergenerational therapeutic relationship. Overall, the results shed light on various intergenerational transference phenomena or roles in which young therapists are perceived. Furthermore, the results show a tendency to seek harmony exhibited by older patients, which was reflected in their behaviour towards young therapists. In the following, these two main findings will be discussed further.

### Transference phenomena

Our findings showed that the respective transference phenomena (including perceived roles) were linked to individual experiences, desires, and fantasies experienced by older patients, which were transferred to young therapists. According to Bollas (p. 933) [24], transference can be understood as the unconscious desires or re-experienced memories of old relationships with previous objects that are projected onto the therapist. Specifically, our results revealed that older patients transfer desires for a close relationship with their grandchild, fantasies about an idealised child, or longing for a partner onto young therapists. For example, Rosie felt that her young therapist reminded her of her beloved granddaughter, Martha seemed to see in her young therapist an idealised son and projected desires for a grandchild, while Richard fantasised his young therapist as a partner and felt amorous feelings. Staats [25] described models of the therapeutic work in terms of different roles that therapists can take, such as buddy/confidante, supporter, detective, nurturing mother or father, professional/expert, teacher, trainer or sparring partner.

On the one hand, these transferences can lead to a positive and productive therapeutic relationship (cf. [26]), while on the other hand, certain particularities can occur, such as older patients taking on a supportive (grand)parental role or having difficulties talking about sexuality. When fantasies and feelings linked to negative experiences, such as abuse, are transferred to young therapists, negative transferences may result in the termination of therapy if they are not recognised and addressed. In the case of Daisy, the decision to end the therapy might have been driven by a negative transference or a certain role attribution. Perhaps, the young male therapist evoked associations of the experienced sexual abuse, potentially tied to a similar age of the abuser. Establishing trust with her therapist posed a challenge for Daisy, especially since she felt that he did not believe in her abuse. In this context, it is important to mention that it can be challenging for young therapists to deal with the devaluation of older patients, e.g., regarding their competencies [10]. In summary, the young age of the therapist might act as a trigger for positive and negative transferences or the attribution of specific roles on the part of the older patient.

The analysis showed that the age difference was reflected in the identified transferences. Most notably, our results indicated a child transference, a grandchild transference, a sibling transference, an erotic love transference, and a perception of the therapist as an authority figure (cf. [27,28]). The latter could either relate to a parental transference or can be associated with a tendency to seek harmony and avoid conflict by recognising authority. Our results echo Hiatt [29], who pointed to a multigenerational transference among older patients and distinguished a child-grandchild, sibling, and parental transference. Additionally, Radebold [30] described the transference phenomenon of an inverted transference where older patients transfer inner images of real or fantasised (grand)children onto young therapists. Our results provide some evidence for the occurrence of such an inverted transference, exemplified by the cases of Rosie and Martha.

#### Specific features of transference

It was important to many older participants to be taken seriously and not to feel old when around young therapists. For example, some older patients emphasised that it was important for them to be treated “as a well-rounded and valuable individual” and “not just written off as a granddad”. On one hand, these remarks could be linked to negative views on aging and self-perceptions of the older patients, as well as negative societal perceptions of old age [31]. The presence of such pessimistic self-view and self-deprecating tendencies among older patients might also correlate with symptoms related to depressive psychological disorders [32].

Older patients might become particularly aware of their more advanced age due to the direct contrast between themselves and their young therapists. By taking on an admiring and supportive parental or grandparental role, negative feelings, such as envy and aggression, could possibly be reduced through a reaction formation. Baumeister et al. (p. 1085) [33] explain the concept of reaction formation as transforming “a socially unacceptable impulse into its opposite” to protect the self-esteem by engaging in a behaviour that portrays a contrary trait to the actual undesirable trait. For instance, indications of hostility or intolerance could be met by demonstrating oneself as peace-loving and tolerant. We argue that a child and grandchild transference can therefore serve to stabilise self-esteem, thereby strengthening feelings of self-worth and self-efficacy (cf. [34]). For instance, many older patients described the feeling of wanting to support young therapists.

For older patients, the psychosocial developmental tasks of shaping the next generation (generativity) and accepting the life cycle (integrity) come to the fore [35]. In keeping with generativity, older patients can pass something on to young therapists and avoid dealing with old age and death by unconsciously assuming the role of a parent. Erotic transference and feelings of being in love, as in the case of Richard, might also serve as a form of defence, for instance, to cope with the fear of death or feelings of loneliness by fantasising about a romantic relationship with a young therapist or as a means to maintain power and control of the therapeutic relationship (cf. [36]).

Our results show that positive child and grandchild transferences build trust and can diminish initial fear of entering psychotherapy in older patients. For example, one older patient described feeling less inhibited with young therapists than with older therapists. In general, an interesting contradiction emerged, as most older patients stressed how competent their young therapists were, all while feeling a desire to help them. These results can be linked to a transference dynamic where older patients place their young therapists in the role of their (grand)children and support them. This might enable older patients to maintain influence and preserve their competence. Thus, showing protective behaviour can also be traced back to uncertainty about what young therapists (perceived as children) can endure. For instance, Daisy had challenges in opening up about her traumatic experiences to young therapists (as well as her own children) compared to therapists of an older age.

Another finding concerned how older patients and young therapists dealt with the issue of sexuality. DeLamater and Sill [37,38] suggested that sexual interest remains moderate to high among most women and men in their 70s. However, our results indicated that especially those patients who reported having a child or grandchild transference, did not want to address their sexuality with young therapists. Humboldt et al. [39] identified challenges older adults feel regarding their sexual well-being, as shared in therapy, such as partner unavailability, family issues and physical changes due to aging. In this context, it is important to mention that older patients might feel inhibitions regarding discussions about their sexuality with their health professionals in general. As an illustration, Rosie explained that her therapist’s young age was an “impediment” to talking about her sexuality. Additionally, Martha also expressed her reluctance to address the subject of sexuality with her young therapist. Both patients drew a parallel to their reservations about discussing sexuality with their (grand-)child. This feeling of inhibition could be explained by a general taboo concerning speaking to (grand)children about sexuality. Sharing sexual problems with young therapists could also trigger unconscious feelings of competition and envy leading to avoidance being used as a defence mechanism. On the other hand, it could also be difficult for young therapists to address sexuality with older patients, e.g., for fear of rejection or interpersonal conflict, and Hillman [40] pointed out that negative countertransference, including fear, confusion, contempt, and denial, is usual among young therapists.

### Seeking harmony as a defence mechanism

Our results demonstrate that the older patients exhibited a tendency to seek harmony and described the therapeutic relationships as predominantly harmonious. Our findings revealed that older patients displayed friendly and supportive behaviour towards young therapists as well as conflict avoidance regarding uncomfortable issues and interpersonal conflicts. We see the tendency to seek harmony as an unconscious defence against dealing with conflicts, with older patients preferring to maintain harmonious therapeutic relationships with young therapists instead. From a psychodynamic perspective, psychological defence mechanisms primarily serve as a protective function, helping the individual to cope with the challenges of reality and to avoid unpleasant, threatening, painful or distressing situations. However, consciously dealing with sensitive issues can be fundamental for personal development. Defence mechanisms might become dysfunctional and impede this crucial processing, reducing overall well-being. Young therapists have to reflect on the function of a defence mechanism versus its potential harm for personal development.

Older patients might infantilise young therapists and support or protect them as a defence mechanism, which reveals a link between defence, harmonious relationships, and transference, i.e., young therapists are placed in the role of (grand)children whom older patients care for. In general, our results indicate that older patients feel the need to be taken seriously and treated as equals, but also wish to be spared from negative experiences and not have to deal with any more conflicts. For instance, Rosie compared the positive reinforcement and support she received from her young therapist to prior therapies involving conflicts and confrontation. She emphasised the importance of enjoying her remaining time and wanted to avoid further conflicts. Concurrently, Rosie stressed the significance of being taken seriously and being treated as an equal.

This finding raises the question of what this contradiction means for a psychodynamic conflict-centred treatment, which focuses on the formulation of internal conflicts. Psychodynamic therapy follows the assumption that “psychological disorders are rooted in conflicting motivational states, often unconscious, which the individual responds to with a variety of habitual strategies (psychiatric symptoms).” (p. 245) [41]. Thus, the understanding and processing of underlying intrapsychic as well as interpersonal conflicts can relieve these symptoms. However, older patients might show a more pronounced conflict avoidance regarding uncomfortable unconscious contents, while simultaneously wanting to be taken seriously. To be taken seriously should, in our understanding, also involve being confronted with internal conflicts to contribute to a long-term improvement of the symptoms. If such confrontation is less likely and potentially more difficult with older patients, then therapists will have to find ways to address negative issues sensitively and reasonably in psychotherapy. This might be particularly challenging for younger therapists with older patients.

Additionally, it should be taken into account that the socialisation of older patients, e.g., in terms of avoiding open criticism (especially in the GDR), can also play a role in more harmonious behaviour and therapeutic interaction.

Most older patients reported having had few to no conflicts with young therapists. However, some interviews suggested signs of conflictual tension despite older patients vehemently denying it. This finding can be interpreted in light of increasing conflict avoidance in old age. According to socio-emotional selectivity theory [42], the need for emotional well-being increases with age as people become aware of the finite nature of their own lives. This results in personal priorities shifting, leading older people to focus on a few harmonious relationships and positive social experiences [43]. From this perspective, the avoidance of conflicts by older individuals may be viewed as a strategy to attain emotional goals, such as improved well-being and harmonious relationships. Birditt and Fingerman [44] also showed that older adults react to social tension with more avoidance and fewer confrontation strategies than younger people. These assumptions align with our finding that some older patients showed a tendency to avoid conflict and instead focus on positive aspects. Oberhauser et al. (2017) [45] understand conflict avoidance in old age as an adaptive reaction to protect social relations and to deal with feared loneliness.

Moreover, a harmonious therapeutic relationship could also be explained by a conflict avoidance of young therapists. For example, Violet recounted that her young therapist would sometimes let go of a critical topic and return to a harmonious setting in the next session again. Moreover, some older patients fantasised about discontinuing the therapy when confronted with potential conflicts to which young therapists might respond through countertransference reactions. Peters et al. [46] showed that psychoanalysts considered friendly therapeutic behaviour more important for older patients in comparison to younger patients. Relatedly, Boschann et al. [10] noted that young therapists behaved in a more friendly, cautious, and less confrontational manner with older patients. Specifically, young therapists were less likely to confront older patients with negative issues in order to spare and protect them, or they behaved more respectfully due to deeply rooted respect and politeness norms. Thus, we assume that a particularly harmonious therapeutic relationship dynamic might evolve as a result from a friendly and less confrontational behaviour of young therapists and the conflict avoidance of older patients.

### Limitations and further studies

This study focused on the perspective of older patients, analysing how they described and perceived their personal therapeutic experiences with young psychotherapists. Retrospective self-reports of 12 older patients were used to develop a preliminary grounded theory on transference phenomena and a tendency to seek harmony. Further research is needed to expand on this preliminary theoretical model and to differentiate further and come to clearer distinctions. Given the qualitative nature of this research and the small number of older patients interviewed, the findings are not representative and do not provide conclusions that can be generalised to other countries and cultures. To verify whether our results are specific to the constellation of older patients (G1) and young psychotherapists (G3), further research must be carried out by looking at the experiences made by older patients with older psychotherapists (G1 and G2).

This study covered a wide age range and did not differentiate between age groups above 65 to account for age heterogeneity. Furthermore, we did not specifically assess differences in the therapeutic approach, the therapy setting as well as the potential influence of the interview setting. We concentrated on issues connected with age and thus did not consider aspects concerning gender, culture, and ethnicity. Moreover, the self-selection bias is important to consider here. The older patients who contacted AB to participate in this study felt perhaps more inclined to do so due to their positive experiences with young psychotherapists. Older patients with less positive experiences might not have had the same level of interest to participate. The participating older patients were predominantly positive about psychotherapy and their therapy was likely to be successful. Therefore, more detailed studies should be carried out including older patients who are less motivated to seek therapy or who have terminated therapy in the past. Future studies could use narrative, biographical interviews to shed more light on the link between biographic experiences and transferences. Studying unconscious transference processes based on narratives remains challenging with theoretical and methodical problems (see [47]).

## Implications for clinical training and research

This study provides preliminary insights into how older patients experience and perceive the therapeutic relationship with young psychotherapists. Firstly, the findings differentiate various intergenerational transference phenomena and roles in which the young therapists are perceived. These are associated with certain particularities and challenges, such as talking about sexuality. We believe that psychotherapeutic training institutions can play an important role in raising awareness concerning such transferences and in supporting young psychotherapists in handling them. Our findings can inform young psychotherapists about a potential role reversal which may result in older patients trying to support them. Secondly, the results shed light on older patients’ tendency to seek harmony which can lead to overly harmonious therapeutic relationships in which conflicts are avoided as part of an unconscious defence. We believe that this is crucial information for young psychotherapists who work with a psychodynamic conflict-centred as well as CBT approaches. When starting to work with older patients, it can be beneficial for young psychotherapists to clarify that conflicts are an integral part of the therapeutic process rather than obstacles. Dealing with conflicts, whether internal or interpersonal, can be an opportunity for patients to enhance self-awareness. By communicating that the therapeutic setting provides a safe and supportive environment in which conflicts can be experienced and resolved, young psychotherapists may aid older patients in moving beyond conflict avoidance and towards a greater understanding their underlying patterns of thought and behaviour. In general, our findings may help young psychotherapists to better understand the intergenerational therapeutic relationship, including transference dynamics, attributed roles, and behavioural patterns of older patients. We assume that aspects of our findings are of interest for young therapists regardless of their theoretical orientation.

## Supporting information

S1 File Interview protocol. (PDF)
