# Supplementary material for: Older patients’ perspectives on the therapeutic relationship with young psychotherapists
Source: PLoS One. 2024 May 14;19(5):e0295834. doi: 10.1371/journal.pone.0295834 (PMC11093373; doi:10.1371/journal.pone.0295834)
Supplement: S1 File — (PDF) [file pone.0295834.s001.pdf]

S1 File. Interview protocol.

General information

Interviewer:	______________________________
Participant no.:	______________________________
Interview duration:	______________________________
Post interview comments:	______________________________

Introductory protocol

Thank you for your interest in this study. The participation in this study is voluntary and you may stop the interview at any time. You may also terminate your participation in this study at any time and without giving reasons, without incurring any disadvantages. The data and personal information collected as part of this study will be treated confidentially. Furthermore, the results of this study will be published anonymously, i.e., your data cannot be assigned to you personally. To facilitate our notetaking, we would like to audio tape the interview. The audio tape will be transcribed, and you can get the transcript if you would like to. The transcript will not be published. Only excerpts will be published and identifying information such as names and places are omitted. To provide anonymity, any personal details will be changed. Please sign the informed written consent for research and recording voices. This statement of consent will be kept separately. Its sole purpose is to be able to prove that you agree to the evaluation in the event of a review by the data protection officer(s). It can no longer be associated with your interview.

Introduction

As a general guide, I have noted some topics and broad questions that I would like to talk about with you. I will open with a topic and might pose further questions as we discuss each topic. I am interested in your personal experiences and perspectives as well as your thoughts and feelings regarding these topics. You are welcome to tell me anything that seems relevant to you on these topics and questions. I would like you to speak as freely and openly as possible. Before we start with the interview, do you have any questions?

Interview guide

Opening question: Thank you for agreeing to this interview. Today we will be talking about your experiences of being treated by a young psychotherapist (aged mid-20 to mid-30). How did you initially come into contact with your psychotherapist(s)?

Dimension: Experiences with psychotherapy

- What are your previous experiences with psychotherapy?
- Why are you seeing your psychotherapist?

Dimension: Therapeutic relationship

- How would you describe the interaction with your psychotherapist?
- Is there an experience with your psychotherapist that you remember in particular?
- *Additional question:* Are there any things you would not discuss with your psychotherapist?
- *Additional question:* Did you experience any interpersonal conflict with your psychotherapist during treatment? (i.e., any kind of argument)

Dimension: Transference phenomena

- How would you describe your psychotherapist?
- Could you please give me an example of how a therapy session between you and your psychotherapist went?

- How do you experience yourself during treatment with your psychotherapist?
- Has your psychotherapist ever reminded you of another person? (e.g., from your past)

Dimension: Opportunities and challenges

- What do you like about working with your psychotherapist?
- What is difficult or challenging for you?
- What recommendation would you give to a young psychotherapist who works with older patients?

Closing question: Finally, is there anything else you think that has not yet been mentioned in the interview or can you think of any additions that are important to you?
